# DLL3-guided therapies in small-cell lung cancer: from antibody-drug conjugate to precision immunotherapy and radioimmunotherapy

**DOI:** 10.1186/s12943-024-02012-z

**Published:** 2024-05-10

**Authors:** Po-Lan Su, Karthik Chakravarthy, Naoki Furuya, Jeremy Brownstein, Jianhua Yu, Meixiao Long, David Carbone, Zihai Li, Kai He

**Affiliations:** 1 Division of Medical Oncology, Department of Internal Medicine, The Ohio State University Comprehensive Cancer Center, 494 Biomedical Research Tower, 460 W 10th Ave., Columbus, OH 43210 USA; 2 Department of Internal Medicine, National Cheng Kung University Hospital, College of Medicine, National Cheng Kung University, 138 Sheng-Li Rd., North District, Tainan, 704 Taiwan; 3 The Pelotonia Institute for Immuno-Oncology, The Ohio State University Comprehensive Cancer Center, 460 W 10th Ave., Columbus, OH 43210 USA; 4 Division of Respiratory Medicine, Department of Internal Medicine, St. Marianna University School of Medicine, Kawasaki, Japan; 5 Department of Radiation Oncology, The Ohio State University Wexner Medical Center, 460 W 10th Ave., Columbus, OH 43210 USA; 6 Department of Hematology & Hematopoietic Cell Transplantation, City of Hope National Medical Center, 1500 E Duarte Rd., Duarte, Los Angeles, CA 91010 USA; 7 Division of Hematology, Department of Internal Medicine, The Ohio State University Comprehensive Cancer Center, 460 W 10th Ave., Columbus, OH 43210 USA

**Keywords:** DLL3, Small-cell lung cancer, Antibody-drug conjugate, T-cell engager, Radioimmunotherapy

## Abstract

DLL3 acts as an inhibitory ligand that downregulates Notch signaling and is upregulated by ASCL1, a transcription factor prevalent in the small-cell lung cancer (SCLC) subtype SCLC-A. Currently, the therapeutic strategies targeting DLL3 are varied, including antibody-drug conjugates (ADCs), bispecific T-cell engagers (BiTEs), and chimeric antigen receptor (CAR) T-cell therapies. Although rovalpituzumab tesirine (Rova-T) showed promise in a phase II study, it failed to produce favorable results in subsequent phase III trials, leading to the cessation of its development. Conversely, DLL3-targeted BiTEs have garnered significant clinical interest. Tarlatamab, for instance, demonstrated enhanced response rates and progression-free survival compared to the standard of care in a phase II trial; its biologics license application (BLA) is currently under US Food and Drug Administration (FDA) review. Numerous ongoing phase III studies aim to further evaluate tarlatamab’s clinical efficacy, alongside the development of novel DLL3-targeted T-cell engagers, both bispecific and trispecific. CAR-T cell therapies targeting DLL3 have recently emerged and are undergoing various preclinical and early-phase clinical studies. Additionally, preclinical studies have shown promising efficacy for DLL3-targeted radiotherapy, which employs β-particle-emitting therapeutic radioisotopes conjugated to DLL3-targeting antibodies. DLL3-targeted therapies hold substantial potential for SCLC management. Future clinical trials will be crucial for comparing treatment outcomes among various approaches and exploring combination therapies to improve patient survival outcomes.

## Correspondence

Small-cell lung cancer (SCLC), which accounts for 13–15% of lung cancer cases, is characterized as an aggressive neuroendocrine carcinoma [1]. It is associated with a dismal prognosis, evidenced by a 5-year survival rate of only 3% in patients with metastatic disease, and a high resistance rate to standard chemotherapy regimens [1]. Despite the advancements in cancer treatment afforded by immune checkpoint inhibitors (ICIs), there is still much room for improvement. For example, one such treatment—the combination of chemotherapy with an anti-PD-L1 antibody (atezolizumab or durvalumab)—offers only a 2-month extension in overall survival (OS) [1]. DLL3, an inhibitory ligand that suppresses Notch signaling [2], has garnered attention as a significant therapeutic target in SCLC. Its relevance is underscored by its high distribution of expression, reaching up to 85%, across various SCLC disease stages and treatment statuses [2]. Transcriptomic analyses further elucidate that DLL3 upregulation is mediated by the transcription factor ASCL1, predominantly expressed in the most common SCLC subtype, SCLC-A [2]. Presently, strategies to target DLL3 are diverse, encompassing approaches such as antibody-drug conjugates (ADCs), bispecific T-cell engagers (BiTEs), and chimeric antigen receptor (CAR) T-cell therapies [3].

Rovalpituzumab tesirine (Rova-T) represents the first-in-class DLL3-targeted ADC for the treatment of extensive-stage (ES) SCLC. It comprises a humanized DLL3-specific IgG1 monoclonal antibody, a pyrrolobenzodiazepine dimer toxin, and a cleavable linker [4]. The phase I study of Rova-T demonstrated an objective response rate (ORR) of 18% in the overall trial population, which increased to 38% in the patients with more than 50% of tumor cells expressing DLL3, with a tolerable safety profile [5]. The subsequent phase II TRINITY study further affirmed the therapeutic efficacy of Rova-T, showing substantial ORRs in SCLC across varying levels of DLL3 expression. Specifically, the study reported ORRs of 14.3% in the DLL3-high (≥ 50% tumor cell-positive) and 13.2% in the DLL3-low (1–49% tumor cell-positive) expression groups [6]. However, Rova-T did not demonstrate superiority over the standard of care in 2 subsequent phase III trials. These included its evaluation as a maintenance therapy post-first-line treatment compared to placebo (MERU study) [7] and as a second-line therapy versus topotecan (TAHOE study) [8]. In the TAHOE study, patients treated with Rova-T exhibited a higher rate of adverse events compared to those observed in phase I clinical trials (approximately 60% versus 38%) [8]. Notable adverse events included severe edema, pleural and pericardial effusion, photosensitivity reactions, and thrombocytopenia. These adverse events are characteristic of those associated with pyrrolobenzodiazepine derivatives and may be attributed to an “early cleavage” of the linker, which could result in the premature release of the payload into the circulation leading to systemic toxicity. Consequently, only about half of the patients were able to complete 2 cycles of Rova-T treatment. Given these discouraging results, further clinical investigations of Rova-T in these trials were discontinued. An emerging antibody-drug conjugate (ADC), ZL-1310, which utilizes camptothecin derivatives as its payload to target topoisomerase I and methylsulfonylpyrimidine tripeptide as cleavable linker [9], is currently under clinical investigation for SCLC.

Tarlatamab (AMG757) represents the first-in-class DLL3-targeted BiTEs. BiTEs are composed of a single-chain variable fragment (scFv) that targets antigens on tumor cells, another scFv that binds to CD3 on T cells, and a fragment crystallizable (Fc) region for extended half-life. DLL3-targeted BiTEs are engineered to link DLL3-positive cancer cells with CD3-positive T-cells, leading to MHC-I-independent T-cell activation and triggering the release of granzyme and perforin, resulting in the lysis of tumor cells [4]. In the phase I DeLLphi-300 study involving pretreated patients with ES-SCLC, of whom 50% were refractory to ICIs, tarlatamab exhibited promising clinical activities. The ORR was 23.4% [10], with median progression-free survival (PFS) of 3.7 months and OS of 13.2 months. Although over half of the patients in the study experienced cytokine release syndrome (CRS), severe toxicity (≥ grade 3) was rare, and all events were reversible without necessitating treatment discontinuation. Immune effector cell-associated neurotoxicity syndrome was another adverse event of note. The phase II DeLLphi-301 study conducted a comparative analysis of the treatment efficacy of tarlatamab at 2 dosage levels, 10 mg versus 100 mg, intravenously every 2 weeks. The study demonstrated that tarlatamab, administered at 10 mg intravenously every 2 weeks, yielded comparable ORRs (40% vs. 32%) and median PFS (4.9 vs. 3.9 months) to those observed at 100 mg intravenously every 2 weeks, with a lower incidence of treatment-related adverse events (TRAEs) (51% vs. 61% for any-grade CRS and 1% vs. 6% for grade 3 CRS) [11]. The current phase III study is being conducted with a regimen of 10 mg administered intravenously every 2 weeks. These data suggested that tarlatamab might represent a breakthrough in treating patients with SCLC who had disease progression over previous chemotherapy and immunotherapy options. The biologics license application (BLA) for tarlatamab has been under review by the US Food and Drug Administration (FDA) since December 2023. The ongoing phase III DeLLphi-304 study will further compare second-line tarlatamab to the standard of care in chemotherapy-pretreated SCLC patients. Concurrently, the phase III DeLLphi-306 study aims to assess the effectiveness of tarlatamab in patients with limited-stage SCLC who have not experienced disease progression following concurrent chemoradiotherapy. In the first-line treatment setting, the DeLLphi-305 study will compare tarlatamab and durvalumab versus durvalumab alone in ES-SCLC following platinum, etoposide, and durvalumab. Additionally, several studies are investigating the synergistic potential of combining tarlatamab with ICIs (Table 1).

**Table 1:** Clinical trials with new treatment strategies targeting DLL3

**Treatment**	**Phase (Patient no.)**	**Setting**	**Primary Endpoint**	**Trial ID**^**p**^	**Status**
ADC					
ZL-1310	I (140)	≥ 2nd line	Safety/MTD	NCT06179069	Ongoing
BiTE					
tarlatamab (AMG757)	I (382)	≥ 2nd line	Safety/MTD	NCT03319940^h^	Ongoing
	II (192)	≥ 2nd line	ORR	NCT05060016^i^	Ongoing
	I (340)	1st line^a^	Safety/MTD	NCT05361395	Ongoing
	Ib (50)	≥ 2nd line^b^	Safety/MTD	NCT04885998	Ongoing
	III (700)	2nd line	OS	NCT05740566^j^	Ongoing
	III (222)	1st line	OS	NCT06211036^k^	Pending
	III (400)	Post CCRT	PFS	NCT06117774^l^	Ongoing
BI764532	I (110)	≥ 2nd line	Safety/MTD	NCT04429087	Ongoing
	I/II (30)	≥ 2nd line^c^	Safety/MTD	NCT05879978	Ongoing
	II (120)	≥ 2nd line	ORR	NCT05882058^m^	Ongoing
	I (44)	≥ 2nd line^d^	Safety/MTD	NCT05990738^n^	Ongoing
	I (60)	1st line^e^	Safety/MTD	NCT06077500^o^	Ongoing
QLS31904	I (290)	≥ 2nd line	Safety/MTD	NCT05461287	Ongoing
TiTE					
HPN328^f^	I/II (57)	≥ 2nd line	Safety/MTD	NCT04471727	Ongoing
RO7616789 (ALPS12)^g^	I (168)	≥ 2nd line	Safety/MTD	NCT05619744	Ongoing
CAR-T					
AMG119	I (6)	≥ 2nd line	Safety/MTD	NCT03392064	Ongoing
LB2102	I (41)	≥ 2nd line	Safety/MTD	NCT05680922	Ongoing
CAR-NK					
NK-92	I (18)	≥ 2nd line	Safety/MTD	NCT05507593	Ongoing

*Abbreviations*: *ADC* Antibody-drug conjugate, *BiTE* Bispecific T-cell engager, *CCRT* Concurrent chemoradiotherapy, *CAR-NK* Chimeric antigen receptor nature killer cell therapy, *CRT-T* Chimeric antigen receptor T-cell therapy, *MTD* Maximal tolerable dosage, *ORR* Objective response rate, *OS* Overall survival, *TiTE* Trispecific T-cell engager

^a^Combination with atezolizumab, with and without carboplatin and etoposide

^b^Combination with AMG404 (anti-PD-1)

^c^Combination with ezabenlimab (anti-PD-1)

^d^Combination with topotecan

^e^Combination with standard of care for SCLC, including chemotherapy and ICIs

^f^albumin as third target

^g^CD137(4-1BB) as third target

^h^DeLLphi-300 study

^i^DeLLphi-301 study

^j^DeLLphi-304 study

^k^DeLLphi-305 study

^l^DeLLphi-306 study

^m^DAREON™-5 study

^n^DAREON™-9 study

^o^DAREON™-8 study

^p^For detailed information, refer to clinicaltrials.gov

There are other DLL3-targeting BiTEs under clinical investigation. These include BI764532 and QLS31904, as well as the trispecific T-cell engager (TiTE) HPN328, which uniquely targets albumin to enhance pharmacokinetics. RO7616789 is also noteworthy for its dual-targeting mechanism, engaging both DLL3 and CD137 (4-1BB) to amplify T cell efficacy (Table 1). The initial outcomes of the phase I clinical trial for BI764532 have shown manageable toxicity levels, evidenced by a low 4% discontinuation due to TRAEs. Effective at doses of 90 μg/kg or above, BI764532 achieved a 25% partial response rate across all participants, with a response rate of 26% in SCLC patients. Furthermore, the median duration of response of BI764532 remains undetermined [12]. Preliminary results from the HPN328 study also showed that 39% of participants undergoing monotherapy observed a decrease in their target lesion size, with this group including 5 SCLC patients. The maximum tolerated dose for HPN328 was not established, and no dose-limiting toxicities or TRAE-related discontinuations were reported [13].

CAR-T cells, engineered to exhibit tumor specificity, have demonstrated significant efficacy in treating patients with hematologic malignancies, thereby revitalizing the field of adoptive cell therapy [14]. Among these, DLL3-targeted CAR-T cell therapy has emerged as a notable area of study. AMG119 represents a class of genetically engineered T-cells expressing an anti-DLL3 binding domain along with the co-stimulatory molecules CD28 and 4-1BB. In a phase I clinical trial involving chemotherapy-pretreated patients with SCLC, AMG119 elicited tumor responses in 1 (20%) patient and stable disease in another, including a complete response in hepatic metastasis, while exhibiting a tolerable adverse effect profile [14]. Other DLL3-targeted CAR-T cell therapies such as LB2102 and ALLO-213 are currently under investigation (Table 1). Complementing CAR-T cell therapy, another phase I trial is exploring CAR-transduced natural killer (NK) cells, notably NK-92 cells, which are equipped with the NKG2D transmembrane domain and the co-stimulatory molecule 2B4-CD3 domain [15]. This configuration potentially enhances the cytotoxic effects of NK cells, offering a promising avenue in cancer therapy (Table 1).

Radiopharmaceutical therapy (RPT) is increasingly recognized as a potentially safe and effective targeted treatment modality for various cancer types [16]. Notably, ^90Y^ibritumomab tiuxetan has been approved for the treatment of non-Hodgkin’s lymphoma [16]. Recent advancements have extended the concept of radioimmunotherapy to SCLC. Tully et al. report on a novel DLL3-targeted therapy, which radiolabeled the anti-DLL3 antibody SC16 with the β-particle-emitting therapeutic radioisotope Lu-177, [^177^Lu]Lu-DTPA-CHX-A”-SC16. This compound delivers precision radiotherapy to cancer cells while sparing normal tissues [16]. In vivo studies using the human SCLC cell line NCI-H82 in immunocompromised mice demonstrated enhanced tumor response and prolonged survival compared to treatment with unmodified anti-DLL3 antibodies. Further evaluation using patient-derived xenograft models confirmed tumor responsiveness at various dosing levels, with only transient hematological and hepatic toxicity observed. The notable efficacy and manageable toxicity profile of this approach warrant its progression into clinical trials. Considering that residual tumors after treatment with [^177^Lu]Lu-DTPA-CHX-A”-SC16 continued to express DLL3 in a preclinical study [16], it would be prudent to investigate the efficacy of repeated dosage of [^177^Lu]Lu-DTPA-CHX-A”-SC16 as well as its combination with other DLL3-targeted therapies, such as DLL3-targeted T-cell engagers or CAR cellular therapies. Moreover, given the well-documented immunomodulatory effects of radiotherapy on the tumor immune microenvironment, particularly regarding macrophages and myeloid-derived suppressor cells [16], exploring synergistic effects with ICIs could be beneficial.

Given that DLL3 is a promising target for SCLC, a pertinent question arises regarding its potential as a predictive biomarker. In the initial human clinical trial of Rova-T, patients with high DLL3 expression in their tumors exhibited better ORRs (35% vs. 0%) and disease control rates (90% vs. 60%) compared to those with low DLL3 expression [5]. However, these observations were not replicated in subsequent phase II and III trials [6, 8]. In the phase I DeLLphi-300 study, there was only a weak association between DLL3 expression levels and treatment response [10]. In contrast, in the phase II DeLLphi-301 study, the response to tarlatamab was observed across patients regardless of DLL3 expression status [11]. These findings indicate that the value of DLL3 expression as a predictive biomarker is still limited. Further studies are warranted to define biomarkers that predict DLL3-targeting treatment response.

In summary, DLL3-targeted therapies have shown enormous promise in managing SCLC. T-cell engagers, especially tarlatamab, have demonstrated improved clinical outcomes and the FDA is currently reviewing the tarlatamab BLA. The other emerging strategies in DLL3-targeted therapy, encompassing both cellular therapy and radioimmunotherapy, have shown promising preclinical results and/or early clinical signals. Additional clinical trials are needed to compare treatment outcomes across different modalities and to evaluate the possibility of combination therapies, potentially enhancing patient survival outcomes.

## Data Availability

No datasets were generated or analysed during the current study.

## Abbreviations

ADC: Antibody-drug conjugate
BiTE: Bispecific T-cell engager
BLA: Biologics license application
CAR-T: Chimeric antigen receptor T-cell therapy
CAR-NK: Chimeric antigen receptor nature killer cell therapy
CCRT: Concurrent chemoradiotherapy
CRS: Cytokine release syndrome
ES-SCLC: Extensive stage small-cell lung cancer
Fc region: Fragment crystallizable region
FDA: US Food and Drug Administration
MTD: Maximal tolerable dosage
NK cell: Natural killer cell
ORR: Objective response rate
OS: Overall survival
PFS: Progression-free survival
Rova-T: Rovalpituzumab tesirine
RPT: Radiopharmaceutical therapy
scFv: Single-chain variable fragment
TiTE: Trispecific T-cell engager
TRAEs: Treatment-related adverse events
